# Economic Hardships in Managing COVID-19 Patients in the Intensive Care Unit: A Retrospective Observational Study at a Tertiary Care Hospital in North India

**DOI:** 10.7759/cureus.54588

**Published:** 2024-02-20

**Authors:** S L Vig, Pooja Goyal, Shipra Saini, Mitasha Singh, Jagdish Prasad, Lokesh Parashar

**Affiliations:** 1 Community Medicine, Employees' State Insurance Corporation Medical College and Hospital, Faridabad, IND; 2 Statistics, Amity University, Jaipur, IND

**Keywords:** pandemic with covid-19, icu, mortality, comorbidities, cost

## Abstract

Background: The information on healthcare expenditure is crucial to know the impact of the pandemic on public health budgets, thereby correctly managing the ongoing crisis and preparing for subsequent waves.

Objective: To estimate the length of stay and cost incurred on COVID-19 patients who died in the ICU.

Methods: It is a record-based descriptive study conducted on 76 deceased COVID-19 patients admitted to the ICU of a dedicated COVID-19 hospital (DCH) between April and October 2020. Central Government Health Services (CGHS) package rate list, Delhi-NCR, was used as a reference for the cost of the ICU bed, ventilator, investigations, and procedures.

Results: The median duration of stay in the hospital was 12 days, and in the ICU, it was eight days. The median total cost of managing the patient was 91,235.6 INR; of this, the median total cost for ICU stay per patient was 6,904 INR. The major proportion of total expenses was contributed by personal protective equipment (PPE) kits, an average of 11,091.33 INR per month. The median cost of stay in the ICU, on the ventilator, in the ward, and mean cost of investigations were higher among those with associated co-morbidities.

Conclusion: Most elderly male with co-morbidities lost their battle after ventilator support in the ICU. Patients with co-morbidities and severe disease not only have a long duration of hospitalization and poor survival rate but also fetch an economic burden close to one lakh on the institute.

## Introduction

The world suffered a major setback when the World Health Organization declared the COVID-19 pandemic on March 11th, 2020. COVID-19 is an acute respiratory illness caused by Coronavirus 2 (SARS-CoV-2) [1]. Since its outbreak in Wuhan, China, in December 2019, the disease has totaled more than 665,003,256 confirmed cases and 6,697.442 deaths worldwide, India's tally shows 44,679,564 cases and 530,705 (1.18%) deaths as of December 31st, 2022 [2].

In addition to the morbidity and mortality, it has caused a tremendous impact on the economy of the nations. The social and economic impact of the disease has already been calculated to be worse than those of the second world war [3]. While major COVID-19-related research efforts are dedicated to understanding its pathophysiology, treatment, prevention, and vaccines, there is a paucity of studies examining the impacts of the pandemic on public health budgets [4]. The information on healthcare expenditure is crucial to knowing the disease burden, correctly managing the ongoing crisis, preparing for the subsequent waves, and guiding the implementation and management of new services such as telemedicine or creating dedicated COVID-19 clinic units [5]. Decision-makers need to understand how their health systems are getting squeezed due to current and future pandemics, particularly to understand the healthcare resource use (e.g., length of hospital stay) and subsequent costs in managing the pandemic [6].

Employment State Insurance Scheme under the legislation of the ESI Act, 1948 is one of the largest social security schemes in India, devised to protect employees and their dependents, covered under it against contingencies such as sickness, maternity, and death or disability due to employment injuries. Medical care under medical benefits is provided through a network of ESI hospitals and dispensaries. The scheme is run solely by the contributions from the employers and the employees as decided from time to time by the Employment State Insurance Corporation. Only insured persons and their families covered under the act get treatment and benefits.

Under the powers vested in section 6(2)(i) of the Disaster Management Act 2005, the National authority had the responsibility for laying down the policies, plans, and guidelines for disaster management (COVID-19 pandemic) for ensuring timely and effective response to disaster [7]. Employment Scheme Insurance Corporation (ESIC) proactively contributed its part in providing the best care to COVID patients by converting twenty-one of its hospitals across India into dedicated COVID-19 hospitals (DCH) and opening its doors to non-insured patients as well as serving its insured persons under ESI Scheme. More than 2400 isolation beds and 550 Intensive care unit (ICU) beds with 200 ventilators have been made available in these hospitals. ESIC Medical College & Hospital, Faridabad, is one such healthcare institution where, besides the COVID-19 testing facility, ICU facility and treatment facility including plasma therapy is also available [8].

Thus, during the pandemic, ESIC, besides providing services to its insured population, was giving medical services to all other non-insured citizens and bearing the expenses out of its own corpus. The current study explored the expenses incurred in managing COVID-19 patients in intensive care units, which can help policy/decision-makers plan resources and budget allocation for future health crises.

Objectives

To estimate the length of stay and cost incurred by COVID-19 patients who died between April and October 2020 at a tertiary care center in the Faridabad district. To determine the effect of co-morbidity and severity of illness on the cost incurred by COVID-19 patients who died between April and October 2020 at a tertiary care center in the Faridabad district.

## Materials and methods

Study design and setting

This record-based descriptive study was conducted in a medical college hospital between March and August 2021 after getting institutional ethics committee (IEC) approval. The state government has declared this setting a dedicated COVID-19 hospital (DCH). A forty-bed ICU exclusively for COVID-19 patients exists to serve moderate to severe COVID-19 patients. The ICU is well equipped with all modern intensive care equipment such as multiparameter monitors and ventilators, specialist medical staff with the availability of full‑time intensivists, 24x7 diagnostic services including radiological investigations, and a pharmacy available round the clock.

Study population

The sampling frame was constituted by the diagnosed COVID admissions in the designated COVID hospital of the district between April and October 2020. The study included hospital records sheets of COVID-19-confirmed deaths in the ICU between April and October 2020, except those who were brought dead. The complete records of the deceased which were considered by the health authorities, were included in the study. The records of indoor ward patients were not accessible or incomplete. Hence, the deaths due to COVID-19 were considered to be our study population. Existing guidelines issued by the government were used to classify the patients into three categories according to the severity of their symptoms. The case definitions of COVID-19 death, mild, moderate, and severe categories were adapted from government guidelines [9,10].

Sample size and sampling technique

Around 2775 cases were admitted to ESIC Hospital, Faridabad, from April through October 2020. Out of these, around 203 patients died in the ICU. This information is taken to assess mortality due to COVID-19, which comes out to be 7.31% at our institute. The minimum sample size was calculated using the following formula: (p=0.073, q=0.927, l=0.06), which was 76 at a 6% margin of error and 95% confidence interval. Systematic random sampling was used to select the records.

Data collection and study variables

Hospital record sheets were used to study the length of hospital stay (ward/ICU) and expenditure incurred in ICU/ward stay (including manpower, equipment, and diet cost), diagnostics (including lab & radiological investigations), and drugs (including supplements) to calculate the total cost in treating the COVID-19 patients. For data collection, a network with a medical record section, nursing staff, and resident doctors was developed to ensure the completeness of records.

The direct cost borne by the health system in managing COVID-19 patients in ICU for the purpose of analysis included bed cost, ventilator, investigations, and procedures (for example, intercostal tube insertion, central venous line insertion, etc.). Since the individual cost was not available for each category, the reference for the costing of the ICU bed, ventilator, investigations, and procedures was taken from the Central Government Health Services (CGHS) package rate list, Delhi-NCR, updated on 22.07.2021 [11]. ESIC follows CGHS rates for ICU referrals of patients from ESIC Hospitals to empaneled private hospital admissions wherever required. It also follows its own rate contract (DG ESIC RC) for drugs published for a defined period for the ESI hospitals by the parent body, i.e., ESIC. The indirect costs involved in COVID-19 management were the cost of PPE kits consumed by healthcare providers visiting the patients admitted to the hospital and the cost of biomedical waste management. The amount of PPE kits consumed was retrieved from the stock register maintained by ICU nursing staff for the period mentioned above. PPE kit consumption per patient/day was difficult to calculate as multiple patients were attended by the same health worker using the same PPE kit, and full bed occupancy was not documented at all times. A rough estimate was computed using the total consumption of PPE kits in the ICU during the study period.

Data and statistical analysis

Data was entered into Microsoft Excel and analyzed using the commercially available statistical software (IBM SPSS V21.0). Continuous data was expressed as mean or median, and categorical data as proportion. The student t-test was applied for continuous variables to test the difference between the two groups. The chi-square test was applied for categorical data to test the difference between groups. Non-parametric tests (Mann-Whitney U test) were applied to test the statistical difference between non-normal distributed variables. Differences were considered to be statistically significant at p-value <0.05.

## Results

The analysis of 76 deceased COVID-19 patients at the tertiary care center shows that the males, 43 (56.6%), outnumbered females, 33 (43.3%). The majority of deaths were in the 61-70 years old age group 23 (30.3%). Three-fourths of 43 (75%) of the patients were in the severe category at the time of admission. Out of this, a majority, 16 (28.1%), were 71-80 years old. Co-morbidities were present in 63 (82.9%) of the study records. Ventilator support was given to 56 (73.7%) of those who died. The median duration of stay in the hospital for the study population was 12 days, {Q1 was 8 days, Q3 was 18 days, and the interquartile range (IQR) was 10 days}, and the median duration of stay in ICU was 8 days, {Q1 was 3 days, Q3 was 13 days and IQR 10 days}.

The median total cost of managing per patient was 91,235.6 INR (IQR- 70,651.4); of this, the median total cost for ICU stay per patient was 6,904 INR (IQR; 8,630 INR) (Table 1).

**Table 1:** COVID-19 severity-wise distribution of study variables * - values in the tables Mann-Whitney U test; # - values in the Chi-Square

	Mild and Moderate COVID-19	Severe COVID-19	Total	p-value
Total N (%)	19 (25.0%)	57 (75.0%)	76 (100.0%)	
Age groups n (%)				
20 – 30	1 (5.3%)	1 (1.8%)	2 (2.6%)	0.47#
31 – 40	1 (5.3%)	0(0.0%)	1 (1.3%)	
41 – 50	1 (5.3%)	6 (10.5%)	7 (9.2%)	
51 – 60	3 (15.8%)	14 (24.6%)	17 (22.4%)	
61 – 70	8 (42.1%)	15 (26.3%)	23 (30.3%)	
71 – 80	4 (21.1%)	16 (28.1%)	20 (26.3%)	
81 - 90	1 (5.3%)	5 (8.8%)	6 (7.9%)	
Gender n (%)				
Male	10 (52.6%)	33 (57.9%)	43 (56.6%)	0.69#
Female	9 (47.4%)	24 (42.1%)	33 (43.4%)	
Associated co-morbidities n (%)				
Yes	15 (78.9%)	48 (84.2%)	63 (82.9%)	0.86#
No	4 (21.1%)	9 (15.8%)	13 (17.1)	
Number of co-morbidities n (%)				
One	11 (57.9%)	15 (26.3%)	26 (34.2%)	0.02#
Two	3 (15.8%)	20 (35.1%)	23 (30.3%)	
Three	1 (5.3%)	12 (21.1%)	13 (17.1%)	
Four	0(0.0%)	1 (1.8%)	1 (1.3%)	
Ventilators stay required n (%)				
No	6 (31.6%)	14 (24.6%)	20 (26.3%)	0.54#
Yes	13 (68.4%)	43 (75.4%)	56 (73.7%)	
Duration of stay on a ventilator (days) Median {Q1, Q3 (IQR)}	2 {0.0, 3 (3)}	2 {0.5, 4 (3.5))	2 {0.0, 4 (4)}	0.56*
Duration of stay in ICU (days) Median {Q1, Q3 (IQR)}	9 {5, 14 (9)}	7 {2.5, 12.5 (10)}	8{3, 13 (10)}	0.39*
Total duration of stay (days) Median {Q1, Q3 (IQR)}	11 {8, 23 (15)}	12 {8.5, 15.5 (7)}	12 {8, 18 (10)}	0.74*

**Figure 1:**
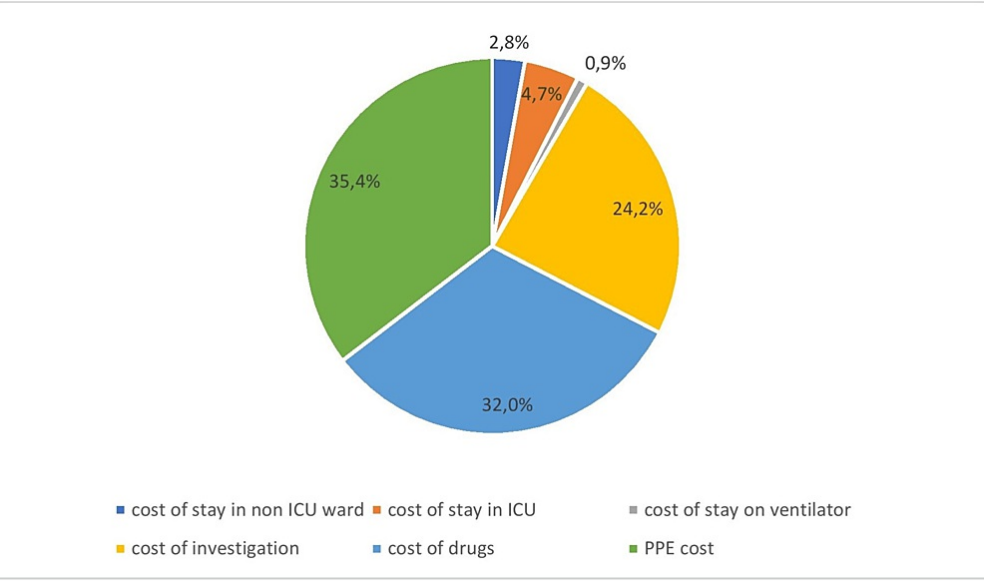
Proportion of total cost under various heads

The cost of stay in the ward was 3,79,000 INR, the cost of stay in ICU was 6,42,072 INR, and the cost of patients on ventilators was 1,22,130 INR. The expenses on drugs and investigation for these patients were 43,75,198 INR and 33,10,835 INR, respectively. Thus, the total cost incurred on the COVID-19 patients during the study period was 882,92,35.95 INR. The total cost incurred on PPE kits used on these patients was taken from the stock register, which came out to be 48,32,852 INR. If it is added to the total cost incurred on COVID-19 patients, it amounts to 136,62,086.95 INR. The major proportion of total expenses was contributed by PPE kits (34.5%), drugs (32.0%), and investigations (24.2%) (Figure 1).

Around three-fourths, i.e. 43 (75.4%), of the severe patients were put on ventilator support, and 20 (35.1%) of the severe COVID-19 patients had two co-morbidities. The median stay in the hospital was higher among severe category patients (median (IQR); 12 (7)) as compared to mild and moderate category (median (IQR); 11 (15)), (p:0.74). The median stay (median (IQR); 12 (7)) in ICU was lower in the severe category as compared to mild-moderate (median (IQR); 9 (9)), (p: 0.39) Table 1. The median total cost was higher among the mild-moderate group (median (IQR); INR 1,00,551.20 (1,11,525.65)) in comparison to the severe category (mean (SD); INR 89,313.80 (44403.00)) (p:0.46) (Table 2).

**Table 2:** Cost of stay, drugs and investigation * - values in the tables Mann-Whitney U test; $ - values in the Independent t-test

	Mild and Moderate COVID-19	Severe COVID-19	Total	p-value
Per patient Cost of stay on ventilator per day (INR) Mean ±SD	1919.77 ± 1430.39	2259.84 ±1966.76	2180.89 ±1849.66	0.57$
Per patient Cost of stay in ICU (INR) Median {Q1, Q3 (IQR)}	7767 {4315, 12082 (7767)}	6041{2157.5, 10787.5 (8630)}	6904 {2589, 11219 (8630)}	0.39*
Per patient Cost of stay in ward (INR) Median {Q1, Q3 (IQR)}	1000 {0.0, 6000 (6000)}	2000 {0.0, 7500, (7500)	2000 {0.0, 7500, (7500)	0.72*
Per patient Investigation cost (INR) Mean ±SD	45,642.63 ±16,918.10	42,870.61 ±16,159.65	43,563.62 ±16,282.93	0.52$
Drugs cost of per patient (INR) Median {Q1, Q3 (IQR)}	50631.20 {18963.42, 111181.46 (92218.04)	31543.34 {10406.65, 77132.79 (66726.14)}	32686.18 {12552, 83059 (70507.0)	0.32*
Total Cost (INR) Median {Q1, Q3 (IQR)}	1,00,551.20 {74384.75, 185910.40 (111525.65)	89,313.80 {75039.77, 119496.77 (44403.00)}	91,235.59 {7498.38, 145637.77} (70,651.39)	0.46*

The median total duration of stay of the deceased without co-morbidities was higher (median (IQR); 12 (9) days) than those with co-morbidities, though the association was not statistically significant (p:0.28). The median cost of stay in the ICU, on the ventilator, in the ward, and mean cost of investigations were higher among those with associated co-morbidities. The median cost of drugs used for the study population was higher for those without co-morbidities (median (IQR); 50,631.2 (129956.25)) as compared to those with co-morbidities (median (IQR); 89313.0 (59413.3)), (p:0.13). The median total cost was lower among those with co-morbidities, although statistically non-significant (p:0.34) (Table 3).

**Table 3:** Presence of associated comorbidity-wise duration of stay and cost distribution * - values in the tables Mann-Whitney U test; $ - values in the Independent t-test

	Presence of Associated Co-morbidities			
	Yes (63)	No (13)	Total (76)	p-value*
Ventilator support is required	47 (83.9%)	9 (16.1%)	56 (100%)	0.73
Ventilator support is not required	16 (80.0%)	4 (20.0%)	20 (100%)	
Total duration of stay (days)- Median (IQR)	12 {9, 18 (9)}	9 {4.5, 14.5 (10)}	12 {8, 18 (10)}	0.28
Duration of stay in ICU (days)- Median {Q1, Q3 (IQR)}	8 {3, 13 (10)}	7 {2, 11 (9)}	8 {3, 13 (10)}	0.47
Duration of stay on the ventilator (days)- Median {Q1, Q3 (IQR)}	2 {0.0, 4 (4)}	1 {0.0, 7 (7)}	2 {0.0, 4 (4)}	0.86
Per patient Cost of stay on ventilator per day (INR)- (Mean ±SD)	2124.00± 1846.09	2478.00± 1951.02	2180.89 ±1849.66	0.60$
Per patient Cost of stay in ICU (INR)- Median {Q1, Q3 (IQR)}	6904.0 {2589, 11219 (8630.0)}	6041.0 {1726, 9493 (7767.0)}	6904 {2589, 11219 (8630)}	0.47
Per patient Cost of stay in ward (INR)- Median {Q1, Q3 (IQR)}	2000 {0.0, 7000 (7000)}	0 {0.0, 7000 (7000)}	2000 {0.0, 7500 (7500)}	0.30
Per patient Investigation cost (INR)- Mean ±SD	44,392.62 ±16,632.55	39,546.15 ±14,376.27	43,563.62 ±16,282.93	0.29$
Per patient Drugs cost (INR)- Median {Q1, Q3 (IQR)}	29,506.45 {11371.55, 75834.98 (64,463.43)}	50,631.2 {22012.14, 151968.3 9(1,29,956.25)}	32686.18 {12552, 83059 (70507.0)}	0.13
Per patient Total cost (INR)- Median {Q1, Q3 (IQR)}	89,313.0 {74879 134292.3(59,413.3)}	1,00,551.2 {77326.2, 210056.4 (1,32,730.2)}	91,235.59 {74986.38, 145637.77(70,651.39)}	0.34

## Discussion

The present study was conducted in a tertiary care hospital and associated with a medical college. The death audit was conducted using standard COVID‑19 death audit proforma to find the cause of death [12], wherein it was found that most of the cases were of severe category and died in the ICU. The median duration of stay in the current study was 12 days, and in ICU was 10 days. Rees et al., in their systematic review of the length of stay of COVID-19 patients, reported that the median stay in ICU ranged from 5 to 19 days [13].

The total cost incurred by the COVID-19 patients during the study period was 882,92,35.95 INR. On average, the total per-patient cost of care for COVID-19 cases in the study setting comes to 1,16,174.14 INR. The average duration of stay in the hospital was 14.78. The cost of investigation (average 43,563.62 INR per patient) and drugs (32,686.18 INR per patient) are the main contributors to the total cost. This could be because of the increased number of special investigations like HRCT chest, IL-6, D-Dimer, LDH, CRP, Procalcitonin, Ferritin, and other blood gas analyses to monitor oxygen saturation. However, this amount is lower than actual as the PPE cost could not be calculated for individual patients.

In the present study, though limited to ICU admission wherein the outcome was death, the median cost of stay of one day in ICU was 6041 INR per patient, and for non-ICU ward stay, it was 6000 INR. This was lower than the rates fixed by the State government of Haryana for private hospitals to reduce disparity in charges across various private hospitals so that inpatient care became equally accessible to all [14]. It could also be because the rates we have taken are for CGHS packages, the drug cost as per the existing rate contract of ESIC, and the actual cost incurred on the procurement of non-RC drugs. A study done by Reddy et al. found that the maximum cost was incurred by hospital drugs and disposables, bed charges, equipment charges, bio-safety protective gear (PPE), and pathological and radiological tests in COVID-19 management [15]. Similar findings were found in our study, too. In a study by Agrawal A et al. on duration and cost of stay in a medicine ICU from a tertiary care center in Mumbai, the average cost of a stay in ICU per patient was reported to be 3454 INR and the average cost of stay per patient per day was 842.4 INR. The average total expenditure per patient in the above study was 27,213 INR, and the average cost per day per patient amounted to 6637.3 INR. This was much lower than the cost of treatment of deceased COVID-19 patients in the current study [16]. Another study done by Kumar P et al. to estimate the per bed per day cost of delivery of health care in polytrauma and single-specialty ICUs in an apex trauma care facility in India reported multi-specialty ICU cost being Rs. 14,977/bed/day and the neurosurgery ICU with Rs. 14,307/bed/day [17]. The reason for such high cost could be that the apex institutes of the country have a maximum number of nursing hours per patient in their ICUs as compared to any other public sector hospitals, and also, the cost of consumables in neurosurgery ICU is higher compared to general ICU. Shelat PR et al., in their costing analysis from a private hospital in India, reported the mean ward bed per patient cost as 6815 INR, the mean ICU bed cost was 15,325 INR, and per patient mean ventilator cost was 6450 INR. Per the patient, the current study's ventilator stay cost was 2180.89 INR, which was lower [18]. The current setting, being a public sector unit with central government rates applied, justifies the lower rates of ventilator charges.

A comparison was also attempted to see the variation in cost among the patients admitted with co-morbidities and without co-morbidities. The cost of care in the ICU for the patients with co-morbidities is slightly lower than for the patients without any associated co-morbidities. This is because certain high-cost drugs like Ramdesivir were given to patients without any co-morbidities as per the protocol [19]. Since literature is scarce, a comparison with other centers was limiting. There was no statistical difference between the length of stay of patients with and without co-morbidities or with different severity of COVID-19 at ICU. It has been observed in previous studies that predictions for survival in the ICU are greater than in the ward in selected patients. The severe COVID-19 patients in the current study stayed for fewer days in the ICU than mild-moderate patients. Although the outcome was the same in both categories, COVID-19 patients in the moderate category had a longer stay in the ICUE due to their disease progression to the severe category and, ultimately, death.

Limitations

Expenditure on patients admitted to ICU requiring high-cost drugs and/or investigations represents only the tip of the hospital expenditures. Invisible costs in the form of expenditures on transport, food, time, etc., borne by the patient’s family were not accounted for. The other obstacle the author faced was calculating the cost of a PPE kit per patient, as one healthcare provider was using a single PPE kit for collectively managing patients, irrespective of the number of patients admitted at that time. Further, only deceased COVID-19 patients were included in the analysis; hence, the further cost of managing ICU patients who were cured can be taken up for future research.

## Conclusions

It is to be reiterated that the above economic burden of deceased patients was borne by the ESIC, whose funds are contributed by employers and insured employees of factories. ESIC is mandated to treat insured patients only, but there was no discrimination in disastrous situations like this. The result of this study can help in the resource allocation for ventilators, oxygen, ICU beds, and budget for managing such pandemic situations where the health system feels stressed and stretched.

Mostly, elderly male with co-morbidities lost their battle after ventilator support in the ICU. Patients with co-morbidities and severe disease not only have a long duration of hospitalization and poor survival rate but also fetch an economic burden close to one lakh on the institute.
